# Reduced Fertility in Patients' Families Is Consistent with the Sexual Selection Model of Schizophrenia and Schizotypy

**DOI:** 10.1371/journal.pone.0016040

**Published:** 2010-12-29

**Authors:** Marco Del Giudice

**Affiliations:** Department of Psychology, Center for Cognitive Science, University of Turin, Torino, Italy; Natural History Museum of Denmark, Centre for GeoGenetics, Denmark

## Abstract

**Background:**

Schizophrenia is a mental disorder marked by an evolutionarily puzzling combination of high heritability, reduced reproductive success, and a remarkably stable prevalence. Recently, it has been proposed that sexual selection may be crucially involved in the evolution of schizophrenia. In the sexual selection model (SSM) of schizophrenia and schizotypy, schizophrenia represents the negative extreme of a sexually selected indicator of genetic fitness and condition. Schizotypal personality traits are hypothesized to increase the sensitivity of the fitness indicator, thus conferring mating advantages on high-fitness individuals but increasing the risk of schizophrenia in low-fitness individuals; the advantages of successful schzotypy would be mediated by enhanced courtship-related traits such as verbal creativity. Thus, schizotypy-increasing alleles would be maintained by sexual selection, and could be selectively neutral or even beneficial, at least in some populations. However, most empirical studies find that the reduction in fertility experienced by schizophrenic patients is not compensated for by increased fertility in their unaffected relatives. This finding has been interpreted as indicating strong negative selection on schizotypy-increasing alleles, and providing evidence against sexual selection on schizotypy.

**Methodology:**

A simple mathematical model is presented, showing that reduced fertility in the families of schizophrenic patients can coexist with selective neutrality of schizotypy-increasing alleles, or even with positive selection on schizotypy in the general population. If the SSM is correct, studies of patients' families can be expected to underestimate the true fertility associated with schizotypy.

**Significance:**

This paper formally demonstrates that reduced fertility in the families of schizophrenic patients does not constitute evidence against sexual selection on schizotypy-increasing alleles. Futhermore, it suggests that the fertility estimates derived from extant studies may be biased to an unknown extent. These results have important implications for the evolutionary genetics of psychosis.

## Introduction

Schizophrenia presents researchers with a complex evolutionary puzzle. The features of schizophrenia include a lifetime prevalence of about 1% worldwide (with substantial between-population variation [1], [2]), a high heritability (85–90% [3]), and substantial negative effects on the reproductive success of affected individuals, especially male patients [4]–[10]. Together, these three features make it difficult to explain how a highly heritable and highly maladaptive phenotype can persist in the population with a remarkably stable prevalence around 1%, which is too high to be explained by a simple pattern of random mutation [11]–[13].

### Sexual selection and the evolution of schizophrenia

In recent years, some researchers have proposed that the evolution of schizotypy and psychosis can be understood in a sexual selection framework. Nettle [14], [15] argued that, although schizophrenia is a disorder with severe maladaptive consequences, psychosis-proneness or *schizotypy* can confer mating advantages on individuals who do not develop a psychiatric condition. More specifically, Nettle proposed that the mating advantages of schizotypy are mediated by increased verbal and artistic creativity, a proposition that has gained empirical support in a number of subsequent studies [16]–[18]. These traits are likely to be especially adaptive in short-term mating contexts; indeed, schizotypy in healthy adults has recently been found to specifically predict increased interest and engagement in short-term mating, but reduced interest and investment in long-term, committed couple relations [19].

Later, Shaner and colleagues [20] advanced a sophisticated theory of schizophrenia based on the biological concept of *fitness indicators*. In their model, schizophrenia represents the negative, maladaptive extreme of a sexually selected fitness indicator, that is, a trait (or suite of correlated traits) that reveals to potential mates an individual's underlying genetic quality (e.g., low deleterious mutation load) and condition (e.g., good nutritional status, low pathogen load). This hypothetical fitness indicator would comprise a number of “verbal courtship” traits including creativity, emotional sensitivity, and expressiveness. It is important to stress that, in the fitness indicator model by Shaner and colleagues, there are at least *two* distinct classes of genetic variants contributing to increased risk for schizophrenia: (a) deleterious mutations in the many brain-expressed genes that contribute to the fitness indicator, which by definition are under negative selection and are maintained by mutation-selection balance; and (b) schizotypy-increasing alleles that enhance the sensitivity of the fitness indicator itself. Schizotypy would then act as an “amplifier” of individual differences in genetic fitness and condition: high-schizotypy individuals would be more likely to achieve outstanding mating success when they have high genetic fitness and/or grow up in good environmental conditions, but also more likely to develop schizophrenia (and to suffer reduced mating success) when they have low genetic fitness and/or grow up in poor conditions. Thus, schizotypy itself can be sexually selected, possibly more strongly so in populations characterized by high levels of mating competition and short-term mating patterns, where displays of good genetic quality are more critical to successful courtship [20].

**Figure 1:**
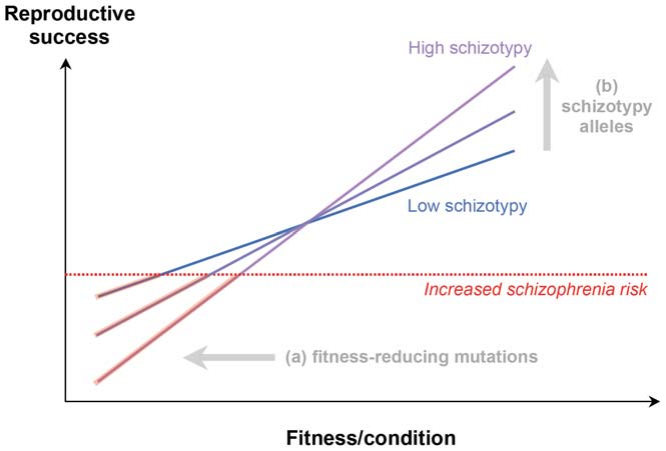
The logic of the sexual selection model (SSM) of schizophrenia and schizotypy. In the SSM, schizotypy enhances the sensitivity of a fitness indicator, by affecting brain processes so as to increase verbal/artistic creativity and other mating-related traits. As a result, schizotypal individuals enjoy higher mating and reproductive success when their genetic fitness is high, but suffer a higher risk of schizophrenia and reduced reproductive success when their genetic fitness is low. The figure shows two classes of genetic factors contributing to increased risk of schizophrenia: (a) fitness-reducing mutations and (b) schizotypy-increasing alleles.

The fitness indicator model by Shaner and colleagues can be easily integrated with Nettle's original proposal: schizotypy-increasing alleles could affect brain processes so as to increase verbal and artistic creativity (together with other mating-related psychological traits), but the outcomes may be either beneficial (mating success) or deleterious (schizophrenia), depending in part on the individual's genetic fitness and condition [15], [21]. The synthesis of the fitness indicator model with the schizotypy-creativity hypothesis can be labeled the *sexual selection model* (SSM) of schizophrenia and schizotypy. Figure 1 provides a schematic illustration of the model. It should be noted that, in the SSM, schizophrenia is caused by a combination of genetic factors (fitness-reducing mutations and schizotypy-increasing alleles) and environmental factors that interfere with developmental processes (thereby worsening the organism's condition); thus, the SSM is not inconsistent with the evidence that environmental factors such as drug use, nutritional deficiencies, and infections can increase the risk of schizophrenia (e.g., [22]–[25]).

### Schizophrenia and fertility

The low fertility observed in families of schizophrenic patients is a potential stumbling block for the SSM. Individuals with a diagnosis of schizophrenia tend to show low reproductive success relative to controls (about .3 to .8 on average); furthermore, the reduction in fertility is more severe in male patients [4]–[10]. Reduced fertility in patients, however, could in principle be compensated for by enhanced fertility in close relatives (parents, siblings and offspring) who share alleles increasing the risk for schizophrenia. Although there have been a few reports of increased fertility in relatives of schizophrenic patients [26], [27], several recent studies converge on the conclusion that fertility in relatives is not high enough to offset the reproductive costs of schizophrenia [5], [8]–[10].

Although these results are statistically robust and based on very large samples, it is still possible that fertility estimates in patients and their relatives are artificially deflated by unrecognized sources of bias. If, for example, schizotypal males had an increased likelihood of extra-pair conceptions, studies based on census and self-report data would easily miss this component of fertility, leading to systematically deflated estimates. This is not an unlikely possibility, given that schizotypal personality traits specifically predict increased engagement in short-term mating and casual sex [19]. As unrecognized extra-pair conceptions specifically deflate male fertility, this may also help explain why the estimated fertility of male patients is consistently lower than that of their female counterparts [4]–[10]. Thus, some of the reproductive benefits of schizotypy may flow from extra-pair copulations, or may have done so in traditional societies. Modern contraception can largely decouple mating success from realized fertility, so that fertility studies carried out in industrialized societies may underestimate the reproductive benefits conferred by schizotypy (or even psychosis itself) over the course of human evolution. Indirect methods have been developed that permit to estimate the number of “potential conceptions” associated with a given number of partners and frequency of copulation [28]; these methods would be extremely helpful in reducing the potential bias associated with extra-pair sexual relationships. To date, however, there are no empirical data supporting the hypothesis that extra-pair copulations contribute to raise fertility in schizophrenic patients; therefore, the results showing reduced fertility in patients' families will be taken at face value in the remainder of the paper.

The finding of reduced fertility in schizophrenic patients' families has been interpreted as indicating strong negative selection on “susceptibility alleles” that increase the risk of schizophrenia. Furthermore, several researchers have argued that reduced fertility in schizophrenics' relatives supports a strict mutation-selection model in which schizotypy-increasing alleles are not maintained nor favored by selection [9], [10], [12]. This interpretation, however, is only partly consistent with the SSM. In the SSM, fitness-reducing mutations are indeed maintained by mutation-selection balance, but schizotypy alleles (which indirectly increase the risk of schizophrenia by increasing the sensitivity of the fitness indicator; see Figure 1) are maintained by sexual selection, and can be selectively neutral (or under balancing selection) in some populations, under negative selection in others, and under positive selection in others still – especially where mating competition is intense and short-term mating is highly prevalent.

In conclusion, the evolutionary implications of reduced fertility in schizophrenic patients' families are not clear in the literature, and many researchers seem to believe that the empirical findings in this area undermine the SSM [9], [10], [12]. However, this assessment may be premature; the alleged inconsistency with the empirical data may be more apparent than real, and the SSM may indeed stand as a plausible candidate for an adaptationist explanation of psychosis.

## Methods

The aim of this paper is to provide a simple mathematical model showing that reduced fertility in the families of schizophrenic patients is fully consistent with the SSM, and can coexist with selective neutrality of schizotypy, or even with positive selection on schizotypy in a given population. It can be shown that, if the assumptions of the SSM hold, empirical studies of patients and their relatives can be expected to underestimate the true fertility associated with schizotypy in the general population.

The argument can be stated verbally as follows. If the SSM is correct, individuals who become schizophrenic patients will tend, on average, to have lower genetic fitness (e.g., more deleterious mutations) and poorer developmental conditions (e.g., nutritional deficiencies, infections, and so on) compared with those who do not. Their close relatives will share not only their schizotypy-increasing alleles, but also their fitness-reducing mutations and their poor environments. Thus, the families of schizophrenic patients will include a disproportionate proportion of low-fitness individuals, who can expected to suffer reduced fertility compared with high-fitness individuals – and especially with those who enjoy outstanding mating success thanks to their schizotypy-increasing alleles. This sampling artifact will result in a downward-biased estimate of the true fertility associated with schizotypy in the whole population. Under some circumstances, such bias can be strong enough to turn a positive selection pressure on schizotypy into a negative one.

A simple but useful mathematical model can be constructed by treating families in a given population as the main unit of analysis. Of course, demarcating families from one another necessarily involves a degree of arbitrariness; however, any reasonable criterion would work for the purpose of this simplified model. Each family can be assigned to one of four classes according to the average level of schizotypy of its members and their average genetic fitness. Families whose members, on average, display comparatively high schizotypy are labeled “schizotypal families”; the remaining families are labeled “non-schizotypal families.” Likewise, “high-fitness families” and “low-fitness families” can be defined as those families whose members' average genetic fitness is (respectively) higher and lower than the population mean. Note that this classification is purely descriptive, and can be applied to any population once families are identified and their members measured on the traits of interest.

## Results

Following Shaner and colleagues [20], [21], we can start by assuming that the probability *P*(*D*) of being diagnosed with schizophrenia is higher against a background of low fitness (*L*) than against a background of high fitness (*H*). For simplicity, let us further assume that (a) in non-schizotypal families, the probability of developing a diagnosable condition is negligibly small; and (b) in the population as a whole as well as in schizotypal families, genetic fitness is symmetrically distributed across families (see [20]), so that 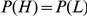.

Let us first consider low-fitness schizotypal families and let *P*(*D|L*) be a family member's probability of being diagnosed with schizophrenia. In high-fitness schizotypal families, a member's probability of receiving a diagnosis is *P*(*D|H*). The relative risk of schizophrenia based on family fitness is thus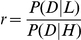. By assumption, 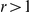.

The probability *P*(*L|D*) that a randomly selected schizophrenic patient comes from a family with low average fitness can be easily found with Bayes' theorem:
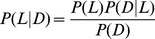
(1)

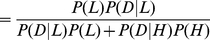
(2)

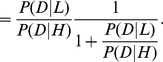
(3)


Substituting *R* in equation 3 gives:
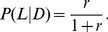
(4)


It follows that:
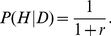
(5)


What happens when fertility in schizophrenic patients and their relatives is used as a proxy for the fertility of schizotypal individuals? Let *W_H_* be the average fertility of members of high-fitness schizotypal families, and *W_L_* the average fertility of members of low-fitness schizotypal families (relative to the population mean). The expected relative fertility *W* of a random member of a schizotypal family is then:
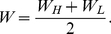
(6)


However, when only diagnosed patients and their relatives are sampled, the expected proportion of high- versus low-fitness families in the sample will no longer reflect that in the population, as low-fitness families will be over-represented (eq. 4) and high-fitness families will be under-represented (eq. 5). Since, on average, members of low-fitness families (both patients and their relatives) are expected to suffer a decrease in fertility because of diminished mating success, the disproportionate inclusion of low-fitness families will lead to a negative bias on estimated fertility.

In particular, the estimated relative fertility 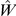 will be:
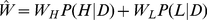
(7)

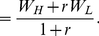
(8)


Let *d* be the difference 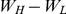. In the SSM, individual differences in genetic fitness are translated into differences in mating success and fertility; thus, 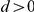. The difference between the true and estimated relative fertility of members of schizotypal families is:
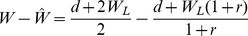
(9)

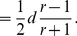
(10)


Since 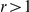 and 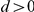, the difference is always positive, i.e., the true fertility associated with schizotypy is going to be consistently underestimated. In some cases, this underestimation bias may result in 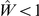 (indicating negative selection on schizotypy) even when 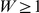 (that is, when schizotypy is selectively neutral or under positive selection). Specifically, 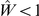 even when 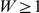 if:
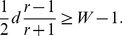
(11)


For example, imagine a population where the relative risk of schizophrenia in low- versus high-fitness schizotypal families is *r* = 3, and the overall difference in relative fertility between members of high- and low-fitness schizotypal families is *d* = 1 (not an unrealistic estimate, considering that *d* also includes the fertility-reducing effects of schizophrenia). In this case,
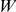
will be underestimated by .25 Thus, a relative fertility 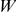 = 1 (indicating selective neutrality) would result in an estimated 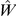 = .75, wrongly suggesting strong negative selection on schizotypy.

In summary, if the SSM is correct, it follows that studies of schizophrenic patients and their relatives consistently underestimate the true fertility associated with schizotypy in the general population (eq. 10). This underestimation bias can lead to infer negative selection even when schizotypy is in fact selectively neutral (or under balancing selection), or even under positive selection (eq. 11). Moreover, equation 10 shows that the underestimation bias becomes more severe with higher values of *d* and *r*; but high values of *d* and *r* are exactly what one expects under the assumptions of the SSM. Indeed, if schizotypy-increasing alleles act as “amplifiers” of differences in fitness and condition, and if schizophrenia corresponds to the low-fitness extreme of a sexually selected fitness indicator, both *d* and *r* should be large, as they directly reflect the hypothesized effects of schizotypy. Although the model presented here is too coarse to permit accurate quantification of the underestimation bias, the qualitative insights it yields should be taken into account in the evolutionary study of psychosis.

## Discussion

In this paper, it was shown that reduced fitness in the families of schizophrenic patients is fully consistent with the sexual selection model (SSM) of schizophrenia and schizotypy; indeed, if the assumptions of the SSM hold, studies of patients' families can be predicted to underestimate the true fertility associated with schizotypy in the general population. In a sense, this result is not completely novel; the consistency of the SSM with low fertility in patients' families was already implicit in the papers by Shaner and colleagues [20] and Nettle and Clegg [15]. However, the lack of an explicit argument clarifying this crucial point has led some researchers to adopt a restrictive view, favoring mutation-selection balance while excluding sexual selection on schizotypy-increasing alleles [9], [10], [12]. The present paper fills this gap by explicitly showing that no contradiction exists between the SSM and reduced fertility in patients' families. According to the SSM, sexual selection is a crucial piece in the evolutionary puzzle of schizophrenia; if this model is correct, mutation-selection balance alone will not be enough to fully explain the persistence of schizophrenia and account for all the available evidence.

In future work on the evolutionary genetics of schizophrenia, it will be crucial to discriminate between two distinct genetic sources of schizophrenia risk: deleterious mutations and schizotypy-increasing alleles. Whereas mutation-selection balance is the most likely explanation for the maintenance of the former, very different selection regimes can apply to the latter [21]. A better understanding of the interplay between these two classes of genetic factors may reveal the logic underlying the persistence of psychosis, and point to the solution of one of the most fascinating puzzles of evolutionary psychology.

## References

[1] Saha S, Chant D, Welham J, McGrath J (2005). A Systematic Review of the Prevalence of Schizophrenia.. PLoS Med.

[2] McGrath J, Saha S, Chant D, Welham J (2008). Schizophrenia: A Concise Overview of Incidence, Prevalence, and Mortality.. http://dx.doi.org/10.1093/epirev/mxn001.

[3] Tandon R, Keshavan MS, Nasrallah HA (2008). Schizophrenia, “just the facts” what we know in 2008. 2. Epidemiology and etiology.. Schizophr Res.

[4] Nanko S, Moridaira J (1993). Reproductive rates in schizophrenic outpatients.. Acta Psychiatr Scand.

[5] Bassett AS, Bury A, Hodgkinson KA, Honer WG (1996). Reproductive fitness in familial schizophrenia.. Schizophr Res.

[6] Nimgaonkar VL (1998). Reduced fertility in schizophrenia: here to stay?. Acta Psychiatr Scand.

[7] McGrath JJ, Hearle J, Jenner L, Plant K, Drummond A (1999). The fertility and fecundity of patients with psychoses.. Acta Psychiatr Scand.

[8] Haukka J, Suvisaari J, Lonnqvist J (2003). Fertility of patients with schizophrenia, their siblings, and the general population: a cohort study from 1950 to 1959 in Finland.. Am J Psychiatry.

[9] MacCabe JH, Koupil I, Leon DA (2009). Lifetime reproductive output over two generations in patients with psychosis and their unaffected siblings: the Uppsala 1915–1929 Birth Cohort Multigenerational Study.. Psychol Med.

[10] Svensson AC, Lichtenstein P, Sandin S, Hultman CM (2007). Fertility of first degree relatives of patients with schizophrenia: A three generation perspective.. Schizophr Res.

[11] Brüne M (2004). Schizophrenia – an evolutionary enigma?. Neurosci Biobehav Rev.

[12] Doi N, Hoshi Y, Itokawa M, Usui C, Yoshikawa T (2009). Persistence Criteria for Susceptibility Genes for Schizophrenia: a Discussion from an Evolutionary Viewpoint.. PLoS ONE.

[13] Huxley J, Mayr E, Osmond H, Hoffer A (1964). Schizophrenia as a genetic morphism.. Nature.

[14] Nettle D (2001). Strong Imagination: Madness, Creativity and Human Nature..

[15] Nettle D, Clegg H (2006). Schizotypy, creativity and mating success in humans.. Proc R Soc Lond B.

[16] Nettle D (2006). Schizotypy and mental health amongst poets, artists and mathematicians.. J Res Pers.

[17] Haselton M, Miller GF (2006). Women's fertility across the cycle increases the short-term attractiveness of creative intelligence compared to wealth.. Hum Nat.

[18] Kinney DK, Richards R, Lowing PA, LeBranc D, Zimbalist ME (2001). Creativity in offspring of schizophrenic and control parents: an adoption study.. Creativity Res J.

[19] Del Giudice M, Angeleri R, Brizio A, Elena MR (2010). The evolution of autistic-like and schizotypal traits: A sexual selection hypothesis.. Front Psychol.

[20] Shaner A, Miller G, Mintz J (2004). Schizophrenia as one extreme of a sexually selected fitness indicator.. Schizophr Res.

[21] Nettle D (2006). Reconciling the mutation-selection balance model with the schizotypy-creativity connection.. Behav Brain Sci.

[22] McGrath J (1999). Hypothesis: Is low prenatal vitamin D a risk- modifying factor for schizophrenia?. Schizophr Res.

[23] Davies G, Welham J, Chant D, Torrey EF, McGrath J (2003). A systematic review and meta-analysis of northern hemisphere season of birth studies in schizophrenia.. Schizophr Bull.

[24] Ferdinand RF, Sondeijker F, van der Ende J, Selten JP, Huizink A (2005). Cannabis use predicts future psychotic symptoms, and vice versa.. Addiction.

[25] Schretlen DJ, Vannorsdall TD, Winicki JM, Mushtaq Y, Hikida T (2010). Neuroanatomic and cognitive abnormalities related to herpes simplex virus type 1 in schizophrenia.. Schizophr Res.

[26] Srinivasan TN, Padmavati R (1997). Fertility and schizophrenia: evidence for increased fertility in the relatives of schizophrenic patients.. Acta Psychiatr Scand.

[27] Avila M, Thaker G, Adami H (2001). Genetic epidemiology and schizophrenia: a study of reproductive fitness.. Schizophr Res.

[28] Pérusse D (1993). Cultural and reproductive success in industrial societies: Testing the relationship at the proximate and ultimate levels.. Behav Brain Sci.

